# Analytical Modeling and Simulation of an Electromagnetic Energy Harvester for Pulsating Fluid Flow in Pipeline

**DOI:** 10.1155/2019/5682517

**Published:** 2019-08-01

**Authors:** Sadia Bakhtiar, Farid Ullah Khan

**Affiliations:** ^1^Department of Mechatronics Engineering, University of Engineering and Technology Peshawar, Peshawar 25000, Pakistan; ^2^Department of Mechanical Engineering, CECOS University, Peshawar 25000, Pakistan

## Abstract

This paper presents the analytical modeling and simulation of an electromagnetic energy harvester (having linear behaviour) that generates power from pulsating fluid flow for pipeline condition monitoring systems. The modeled energy harvester is comprised of a cylindrical permanent magnet and a wound coil attached to a flexible membrane which oscillates due to the pulsating fluid flow in the pipe over which the prototype is considered to be mounted. In the harvester electrical energy is produced due to the relative motion between the coil and magnet. Based on the harvester's architecture a lumped parameter model (single degree of freedom system) is developed and is simulated at different physical operational conditions. The simulation is performed at pressure amplitude of 625 Pa. When subjected to the operational frequency sweep, at the harvester's resonant frequency (500 Hz) and damping ratio of 0.01, the devised model predicted the maximum open circuit voltage of 2.55 V and load voltage of 1.27 V. While operating under resonance, the maximum load voltage of 2.45 V is estimated at load resistance of 100 Ω. However, at an optimum load of 4.3 Ω, the simulation shows a production of 188151.2 *μ*W power at a frequency of 500 Hz.

## 1. Introduction

For the rapid advancement and development of countries the need for energy and natural resources such as water, oil, and natural gas availability is most significant. Mostly the transportation of these resources (water, oil, and gas) is done through pipelines. Protecting the pipeline infrastructure is the foremost priority for the economic growth and progress of every country. Therefore monitoring of pipelines is very necessary in order to avoid any sort of stoppage or wastage of natural resources or the pipeline infrastructure [1]. There are many oil companies in the Gulf countries that completely rely on pipeline network of transportation. This pipeline network is connected to the ports with distant refineries. Various condition monitoring systems are utilized for surveillance of pipeline network.  Table 1 shows the details of monitoring systems for different pipelines in various countries that are functional for purpose of transportation of oil and water [2].

**Figure 1:**
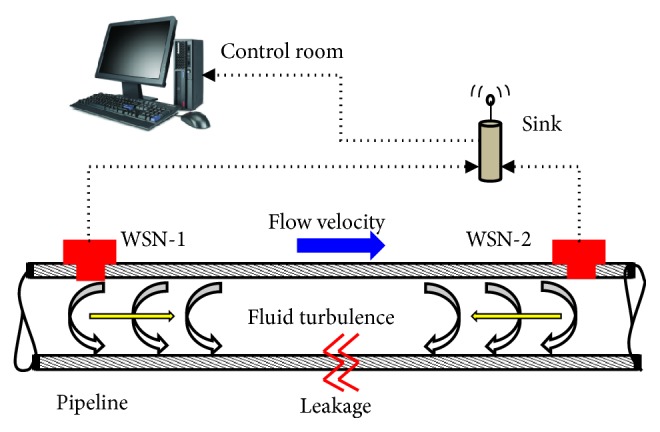
Schematic of pipeline monitoring wireless sensor networks.

There are many technologies used to monitor the pipelines. Most of these technologies depend on a communication network for the transmission of data from the surroundings of the pipelines to the control station [5]. Different parameters are being sensed and monitored for the pipeline monitoring. These parameters could be temperature, pressure, mass flow rate, flow velocity, volume flow rate, etc. In order to monitor the pipeline parameters, Wireless Sensor Networks are used, where the data from pipeline is gathered and transmitted to the distant control station. Such a network consists of a number of detection stations for the purpose of sensing and monitoring; these detection stations are actually the wireless sensor nodes (WSNs) which are multifunctional. These wireless sensor nodes WSNs have become a viable choice for many operations including sensing and supervising of remote systems. The way how WSNs are installed on pipelines for sensing and monitoring purpose is depicted in Figure 1.

**Figure 2:**
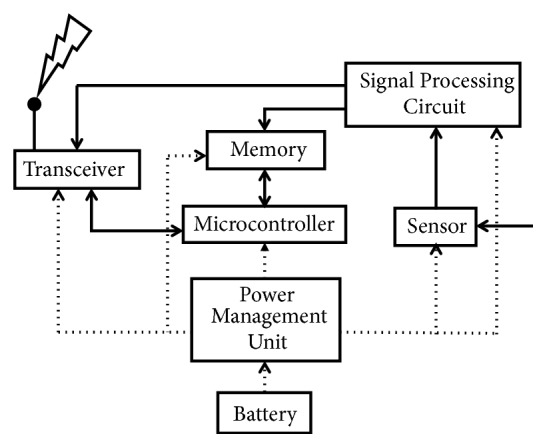
Layout of a wireless sensor node.

The WSN [6] system layout is shown in Figure 2. It consists of a sensor which receives the physical signals; these are sent to the transceiver via signal processing circuit. The transceiver is used for dual purpose receiving the data and transmitting it to the operator. The memory stores the data and generally the overall performance of the system is controlled by the microcontroller. Moreover, the WSN is powered through a battery.

Normally, the batteries are utilized as a power unit for WSNs. Because of restricted lifecycle of a battery and bulky size, they cannot be revived or substituted in remote, deserted, and abandoned areas where the pipelines are passing by. As the WSNs are placed through the immense length of pipeline therefore recharging enormous number of batteries is not feasible. Furthermore batteries are also hazardous to the environment and hence require standard procedures to dispose them off [7]. For long lasting operation of WSNs an alternative source for powering these is a real need. Energy harvesting devices [8] can be used as an alternative solution for power source in WSNs. Such energy harvesters [9] can successfully convert the available ambient energy sources such as wind [10], solar [11], acoustic [12], thermal [13], and mechanical vibrations energy [14] into useful electrical energy [15]. In order to generate electrical energy from vibrations, three basic mechanisms are usually used, that is, piezoelectric [16, 17], electrostatic [18, 19], and electromagnetic [20, 21] transduction. In piezoelectric transduction technique electrical energy is produced by subjecting the piezoelectric material to stress or strain whereas in electrostatic transduction technique when there is a change in the capacitance of the conductive plates (when these move relatively to each other) energy is produced. However, the electromagnetic transduction mechanism utilizes the relative motion between the coil and magnet to generate energy.

Research has been carried out by the researchers to harvest energy that can be later utilized for operating WSNs for pipeline sensing and monitoring systems. Analytical Modeling of EMEH [22] on vibration induced due to generation of Karman Vortex Street in a fluid flow is performed. The harvester consists of a magnet fastened to the diaphragm. The pressure variations in the liquid flow cause the diaphragm to vibrate with respect to the coil, thereby producing electrical energy. Ritz's method is used to determine the deflection of the diaphragm in the harvester. The analytical model for the voltage induced in the loop of the coil is based on Faraday's law of electromagnetic induction. Moreover, a lumped model is developed to analyze the dynamic behavior of the harvester. The magnet is displaced under the maximum and minimum pressure of 3.27 and 2.71 kPa, respectively. The value of the peak-to-peak voltage is nearly 80 mV. D.-A. Wang et al. [23] modeled an EMEH based on flow energy. The prototype model consists of a magnet glued to the diaphragm and a coil is located above the magnet and separated by a small gap. The system is designed such that the liquid variable pressure in the chamber drives the diaphragm attached to the permanent magnet into vibrations. The induced voltage in the coil is determined by Faraday's law of induction. In order to analyze the deflection of the diaphragm, the emf induced in the coil and the pressure distribution within the chamber is obtained by finite element analysis method. Three-dimensional flow analyses are carried out using a commercial software Fluent. The first-order element, CAX4IH, is used for the vibration analyses under the pressure loading. A gage pressure 518 Pa is applied to the diaphragm and the voltage amplitude of nearly 11 mV is achieved. In [24], Wang et al. presented fabrication and modeling for piezoelectric energy harvester based on fluid induced vibrations. The model for estimation of the voltage generated in the piezoelectric film is based on Gauss's law. The charges are accumulated on the piezoelectric electrodes. The values of the pressure considered during simulations are based on the measured pressure levels in the pressure chamber of the fabricated harvester. The simulation shows that the peak-to-peak voltage increases as the pressure difference is increased. The maximum and minimum of the simulated peak-to-peak voltage reported are 1.77 and 2.30 V, respectively, for pressure difference ranges from 1.79 to 2.392 kPa, respectively. A distributed energy harvesting system [25] based on spoiler phenomenon and piezoelectric effect is developed. For a harvester a flow model is simulated and analyzed with ANSYS. Simulation of fluid-solid coupled system and electromechanical coupled system is performed. For the peak-to-peak voltage, open circuit voltage simulations are performed with respect to the flow velocity for different diameters of the bluff body. The maximum peak-to-peak voltage reported is 54 V. E. Bouendeu et al. [26] reported a low-cost vibration based electromagnetic harvester. The open circuit voltage of harvester is expressed in the paper. It also presents the electrical output power of the harvester at the resonance. The overall bandwidth of the electromagnetic vibration harvesters is also given. The excitation frequency by which the harvester delivers the maximum output power is called optimum excitation frequency. It depends upon resonant frequency of the harvester, its mechanical damping, and electrically induced mechanical damping. This dependence is expressed in an equation. Moreover the finite element method (FEM) was used to model and analyze the mechanical resonator of the harvester. F. Khan et al. [27] reported the modeling and simulation for electromagnetic linear EMEHs with a nonuniform magnetic field configuration excited by harmonic vibrations. Models are developed based on Faraday's law of electromagnetic induction and the Lorentz force law. Simulations of the optimized device predicted a load voltage of 90.2 mV and a load power of 40.7 *μ*W at the resonant frequency of 371 Hz.

This paper presents modeling and simulation an electromagnetic linear energy harvester that utilized kinetic energy from the pulsating fluid flow in a pipeline. The generated electrical energy can be utilized for the WSNs used for pipeline sensing and mentoring. In the previous work majority of flow based energy harvesters made use of a bluff body to create vortices in the flow and produce vibrations. However, in this work, a novel architecture for the harvester is adopted that will generate electrical power from the pulsating or turbulent fluid flow that is normally available in the pipe near the inlet and outlet of compressor, blower, and pump; moreover, pipe fittings (tee, elbow, union, valve, flow meters, and pressure gauges) are also responsible for causing fluid turbulence. Moreover, to intentionally produce the turbulent or pulsating fluid flow in pipe a bluff body [22, 28] is put in the flow path, which creates a Karman vortex street (fluid turbulence) which causes the membrane (diaphragm) to vibrate. For the harvester a lumped parameter model (single degree of freedom, spring-mass-damper) and Faraday's law of electromagnetic induction are used to develop an analytical model for voltage and power generation. The analytical solution of the magnetic flux density for a cylindrical magnet is utilized to simulate the magnetic flux density and magnetic flux density gradient over the wound coil and the result is then used to optimize the magnet-coil gap. Moreover, with the devised analytical model, open circuit voltage, load voltage, and load power levels are estimated with respect to excitation frequency, load resistance, damping ratio, and amplitude of pulsating pressure.

## 2. Modeling

**Figure 3:**
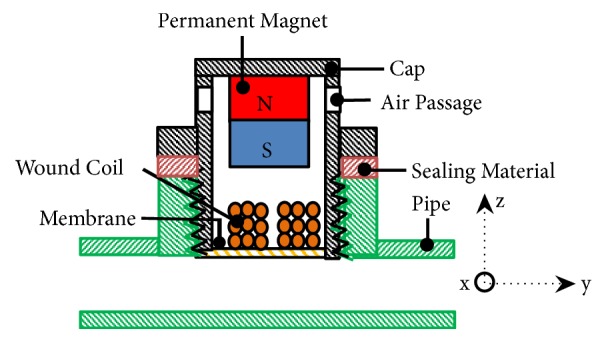
Cross-sectional view of EMEH for pipeline monitoring system.

The schematic of the EMEH for pulsating fluid flow in pipeline is illustrated in Figure 3. The energy harvester is comprised of a wound coil, membrane (steel, rubber, or latex depending on the pressure inside the pipe), permanent magnet, and a threaded casing (cap). Wound coil is attached to the membrane which is bonded to the bottom side of the cap. However, a stationary magnet is fixed to the top inner-side of the cap. The device is supposed to be tightly mounted on the pipe and a packing or sealing material (as shown in Figure 3) is required to provide a perfect fluid sealing and avoid fluid leakage. Due to the pulsating fluid pressure inside the pipe the membrane (coil) oscillates with respect to the magnet and will experience a change in magnetic flux density. The change in magnetic flux will in turn consequently produce an electromotive force (emf) at the terminals of the coil in accordance with Faraday's law of electromagnetic induction. In order to reduce the air damping in the harvester air passages (slots) are provided in the harvester's cap; this will allow easy movement of trapped air in the cap and hence will help in reduction of air compression (air damping) during membrane's vibration. The membrane or diaphragm in the harvester is needed to be designed depending on the pressure levels in the pipeline and as per turbulence amplitudes available at the pipe section. For low pressure pipeline and where amplitude of pressure pulses are minimal, latex membrane is better to be used in the harvester for better performance. For pipelines with moderate pressure levels thick rubber diaphragm is required to be mounted in the harvester; however, for high pressure levels in pipeline a steel diaphragm or plate is to be designed to cope with pressure levels.

**Figure 4:**
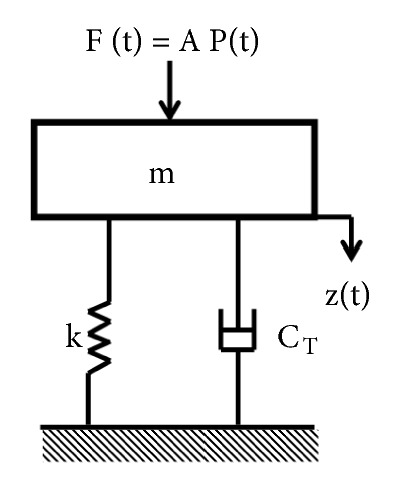
Lumped parameter model of the EMEH.

Figure 4 illustrates the lumped parameter model (single degree of freedom, spring-mass-damper system) of the EMEH. The moving coil has a definite mass (m) and consequently has a kinetic energy. However, the elasticity of the flexible membrane can be modelled as the spring stiffness (k). Moreover, with total damping *ξ*_*T*_ = *ξ*_*M*_ + *ξ*_*E*_, the total energy losses (energy going out of the system) can be represented. For the harvester, the total damping is comprised of mechanical damping *ξ*_*M*_ and electrical damping *ξ*_*E*_. The energy dissipation due to air friction (for air between the magnet and membrane), material interlayer friction (energy dissipated in membrane material), and the energy flow through support (membrane and frame interface) is categorized as mechanical damping. However, the current induced in the wound coil produces a magnetic field which would be opposite to the magnetic field of the magnet (according to Lenz law) causing the electrical damping force. The electrical energy getting out of the system can be modelled as the electrical damping. Furthermore, the pulsating pressure P(t) in the pipeline can cause a pulsating force F(t) = AP(t) acting on a membrane of area A, producing a displacement z(t) in the membrane (coil).

In the harvester, due to the relative displacement between the coil and magnet, the coil experiences the changing magnetic flux* Φ* and voltage is generated in the coil's terminals according to Faraday's law [29]

(1) V=−dΦdt=−ddt∫Bz.dS=−∫ddtBzdsV=−∫dBzdzdzdtds=−U∫dBzdzds

For single layered coil (1) can be simplified as

(2) $$\begin{array}{c} V_{l}=-U{\left(\;\frac{dB_{z}}{dz}\;\right)}_{l}\overset{n}{\underset{i=1}{\sum }}S_{i} \end{array}$$

where *V*_*l*_ is the voltage induced in single layer of a coil and it depends on the relative velocity* U* of the membrane (coil) with respect to the magnet, the magnetic flux density gradient *dB*_*z*_/*dz* over the layer, and the area sum *S*_*i*_ of turns* n* of the layer in the wound coil.

The normal component of the magnetic flux density *B*_z_ from the surface and along a line passing through the center of a cylindrical magnet [29]

(3) $$\begin{array}{c} B_{z}=\frac{B_{r}}{2}\left(\;\frac{z+h_{m}}{\sqrt{{\left(z+h_{m}\right)}^{2}+{r_{m}}^{2}}}-\frac{z}{\sqrt{z^{2}+r_{m}^{2}}}\;\right) \end{array}$$

is determined by the height* h*_*m*_ of the magnet, the remnant flux density* B*_*r*_, the distance* z* from the surface of the magnet to the coil, and radius *r*_m_ of the magnet.

The magnetic flux density gradient

(4) $$\begin{array}{c} \frac{dB_{z}}{dz}=\frac{B_{r}}{2}\left[\;\frac{r_{m}^{2}}{{\left({\left(z+h_{m}\right)}^{2}+r_{m}^{2}\right)}^{3/2}}-\frac{r_{m}^{2}}{{\left(z^{2}+r_{m}^{2}\right)}^{3/2}}\;\right] \end{array}$$

can be determined by taking derivative of* B*_*z*_ with respect to* z*. The magnetic flux density gradient

(5) $$\begin{array}{c} \frac{dB_{z}}{dz}=\overset{N_{L}}{\underset{l=1}{\sum }}{\left(\;\frac{dB_{z}}{dz}\;\right)}_{l} \end{array}$$

has to be computed for number of layers* N*_*L*_ of the wound coil.

**Figure 5:**
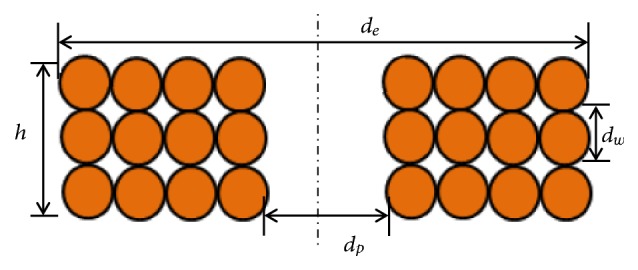
Cross-sectional view of wound coil.

The number of layers for wound coil having wire diameter *d*_*w*_ and height* h *(Figure 5) can be modeled as

(6) $$\begin{array}{c} N_{L}=\frac{h}{d_{w}} \end{array}$$

For a single layer wound coil having* n* number of turns and internal diameter* d*_*p*_, the area sum is estimated as

(7) $$\begin{array}{c} S_{s}=\overset{n}{\underset{i=1}{\sum }}S_{i}=\overset{n}{\underset{i=1}{\sum }}\frac{\pi }{4}{\left(\;d_{i}\;\right)}^{2}=\frac{\pi }{4}\overset{n}{\underset{i=1}{\sum }}{\left(\;\frac{d_{p}}{2}+\left(\;i-1\;\right)d_{w}\;\right)}^{2} \end{array}$$

where

(8) $$\begin{array}{c} d_{i}=\frac{d_{p}}{2}+\left(\;i-1\;\right)d_{w} \end{array}$$

For multiple layers, total voltage

(9) $$\begin{array}{c} V_{T}=U\overset{n}{\underset{i=1}{\sum }}S_{i}\left[\;\overset{N_{L}}{\underset{l=1}{\sum }}{\left(\;\frac{dB_{z}}{dz}\;\right)}_{l}\;\right] \end{array}$$

can be calculated by substituting (5) and (7) in (2).

From the lumped parameter model, the amplitude [30]

(10) $$\begin{array}{c} Z=\frac{z_{o}}{\sqrt{{\left[1-{\left(\omega /\omega_{n}\right)}^{2}\right]}^{2}+{\left[2\xi_{T}\left(\omega /\omega_{n}\right)\right]}^{2}}} \end{array}$$

of displacement of the membrane with respect to the magnet is computed in terms of circular natural frequency *ω*_*n*_, the excitation frequency* ω*, the total damping, that is, sum of mechanical and electrical damping, *ξ*_*T*_ = *ξ*_*M*_ + *ξ*_*E*_, and the static deflection *z*_*o*_ = *F*_*o*_/*k* of the membrane.

In (9), the amplitude of velocity *U* = *Zω* can be obtained as

(11) $$\begin{array}{c} U=\frac{F_{o}\omega_{}}{k\sqrt{{\left[1-{\left(\omega /\omega_{n}\right)}^{2}\right]}^{2}+{\left[2\xi_{T}\left(\omega /\omega_{n}\right)\right]}^{2}}} \end{array}$$

and simplified

(12) UFoωm.k/m1−ω/ωn22+2ξTω/ωn2=fPA2πfn2m1−f/fn22+2ξTf/fn2

in terms of natural frequency *f*_*n*_ and excitation frequency* f*.

When a resistive load* R*_*L*_ is attached to the energy harvester, the load voltage

(13) $$\begin{array}{c} V_{L}=\left(\;\frac{R_{L}}{R_{L}+R_{C}}\;\right)V_{T} \end{array}$$

at the load and load power

(14) $$\begin{array}{c} P_{L}=\frac{V_{L}^{2}}{2R_{L}} \end{array}$$

depends on the load resistance* R*_*L*_ and coil resistance* R*_*C*_.

The coil resistance

(15) $$\begin{array}{c} R_{C}=\frac{\rho_{C}l_{C}}{A_{C}} \end{array}$$

can be obtained with resistivity *ρ*_*C*_ of the copper wire, cross-section area *A*_*C*_ of the coil, and length of the coil *l*_*C*_.

The coil cross-section area

(16) $$\begin{array}{c} A_{C}=\frac{\pi }{4}d_{w}^{2} \end{array}$$

and the coil length for* n *number of turns can be modeled as

(17) $$\begin{array}{c} l_{n}=\pi \left[\;d_{p}+\left(\;2n-1\;\right)d_{w}\;\right] \end{array}$$

For coil's multiple layers,* N*_*L*_ which is the total length of the coil would be

(18) $$\begin{array}{c} l_{C}=N_{L}l_{n} \end{array}$$

Moreover, at resonance (*f = f*_*n*_) the velocity

(19) UresPA4πfnmξT=PA2ωnmξT=PACCξT=PACT=PACE+CM

in terms of electrical damping* C*_*E*_ and mechanical damping* C*_*M*_ can be used to obtain the voltage

(20) $$\begin{array}{c} V_{T_{res}}=U_{res}\overset{n}{\underset{i=1}{\sum }}S_{i}\left[\;\overset{N_{L}}{\underset{l=1}{\sum }}{\left(\;\frac{dB_{z}}{dz}\;\right)}_{l}\;\right] \end{array}$$

levels produced at resonance.

## 3. Simulation

The analytical model developed for the device is simulated according to the parameters and dimensions of the components of the energy harvester as shown in Table 2.

**Figure 6:**
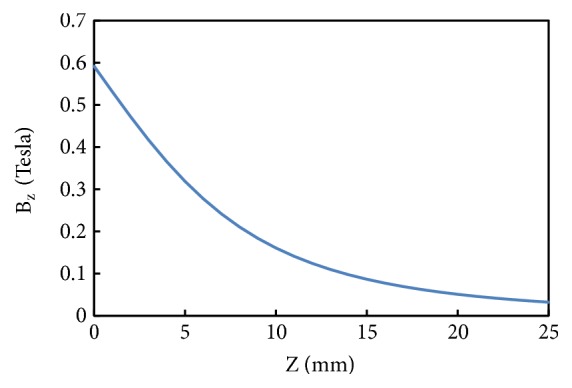
Magnetic flux density vs. distance from the surface of the magnet to the coil.

Figure 6 represents the magnetic flux density of the magnet at different distances of the coil from the surface of the magnet. The results are simulated in accordance with the analytical model for magnetic flux density in (3). As seen, the normal magnetic flux density B_Z_ is 0.59 T from the surface of the magnet. The graph clearly indicates that as the distance between the magnet and coil layers is increased, the magnetic flux density decreases. Therefore, for the better performance of the harvester the gap between the magnet and coil needs to be as small as possible.

**Figure 7:**
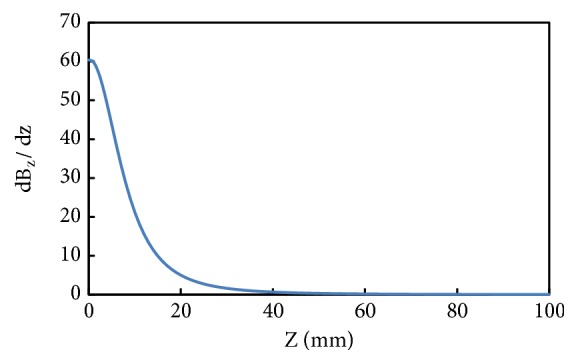
Magnetic flux density gradient vs. distance from the surface of the magnet to the coil.

Similarly, the magnetic flux density gradient versus the distance from surface of magnet to the coil is shown in Figure 7. The simulation result is based on the analytical model in (4). Near the magnet the magnetic flux density gradient is high; however, moving away from the coil the magnetic flux density drastically decreases. The simulation reveals that it is better to have the coil as near as possible to the magnet. However, this gap is also practically constrained by the amplitude of displacement of the coil at resonance. Moreover, from the simulation it can be easily predicted that gap between the magnet and coil should be less than 5 mm for harvester's better performance.

**Figure 8:**
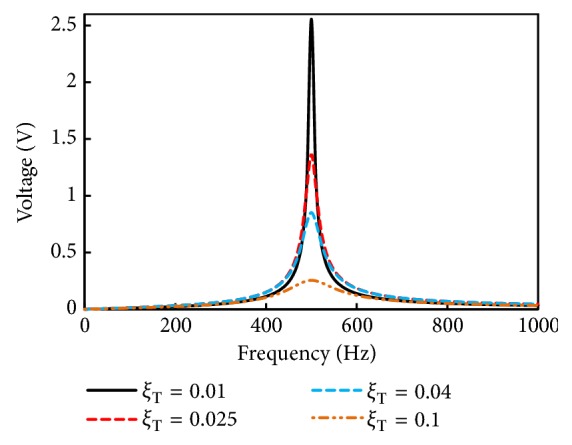
Open circuit output voltage of the simulated harvester at different values of total damping ratio *ξ*_T_.

Figure 8 shows the open circuit frequency response (output voltage) of the simulated harvester at different values of total damping ratios (*ξ*_T_). The open circuit voltage is obtained with (9). In the simulations, the selected range for *ξ*_*T*_ is from 0.01 to 0.1. At the resonance, maximum voltage is produced and moreover as *ξ*_*T*_ is increased the voltage generation at the resonant frequency of 500 Hz is dropped. The simulation shows that a maximum voltage of 2.55 mV is produced at a *ξ*_*T*_ = 0.01.

**Figure 9:**
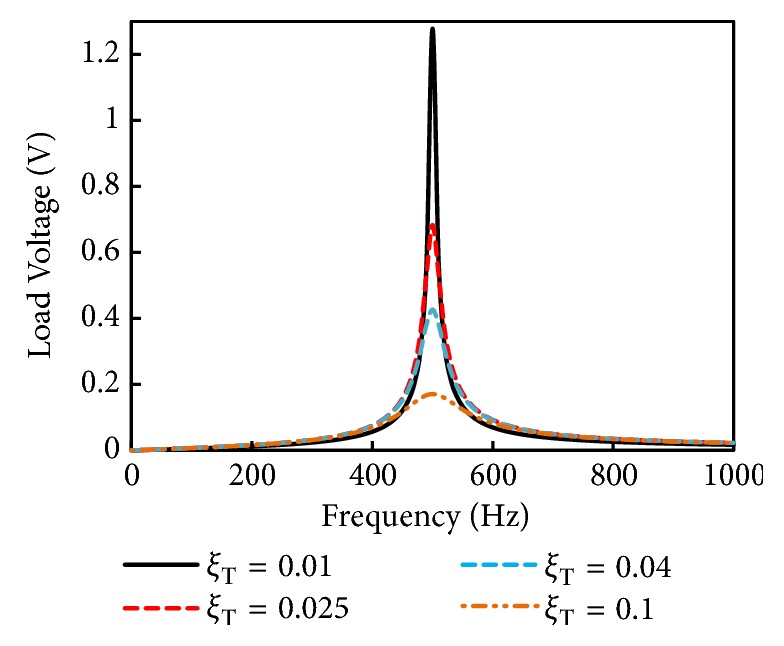
Frequency response of simulated load voltage of the EMEH.

Figure 9 shows the frequency response of simulated load voltage of the EMEH. The load voltage is simulated at different values of total damping ratios (*ξ*_*T*_), the same way as the open voltage is simulated. This simulation is performed with (13). An optimum load resistance, R_L_ = Rc= 4.3 Ω (coil resistance, R_C_ = 4.3 Ω), is supposed to be attached to the harvester and is simulated from 0 to 1000 Hz excitation frequency. At damping ratio of 0.1, 0.04, 0.025, and 0.01, the voltage levels dissipated at the load resistance are predicted to be 0.17, 0.42, 0.68, and 1.27 mV, respectively.

**Figure 10:**
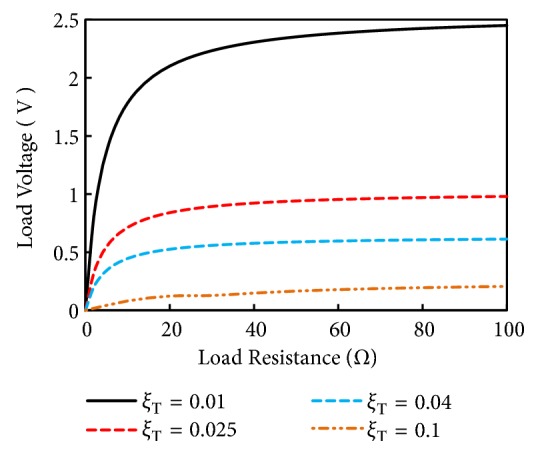
Load voltage generated by the simulated energy harvester.

Figure 10 shows the simulation result of load voltage (13) at resonance as a function load resistance (range from 1 to 100 ohms). As the load resistance is increased the load voltage increases since the current through the resistance drops. Moreover, the load voltage at resistance also enhances as the total damping of the harvester is reduced.

**Figure 11:**
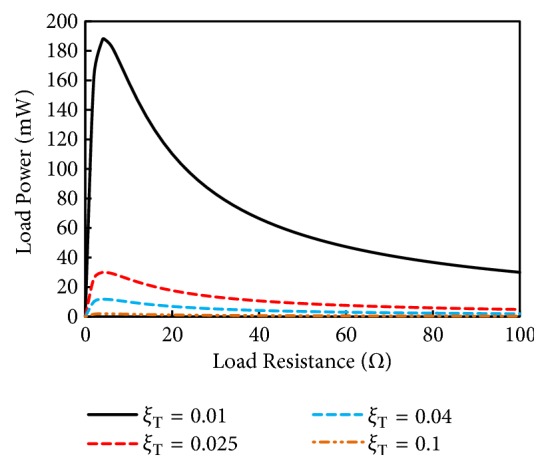
Load power as a function of load resistance produced for the simulated energy harvester.

Figure 11 represents the power produced at resonance as a function of load resistance. Simulation is done for different values of total damping ratios (*ξ*_*T*_) of harvester and is the result of (14). The load power increased as the load resistance is increased from zero. Maximum power is delivered when the load resistance is 4.3 Ω, which is equal to the coil's resistance (load matching condition). Afterwards if the resistance is enhanced the power delivered to the load starts declining sharply. The simulation reveals that, far away from the optimum load, the power delivery to the load (WSN) will be drastically affected. Therefore, in the energy harvesters it is better that harvester's impedance matches the load impedance for better performance.

**Figure 12:**
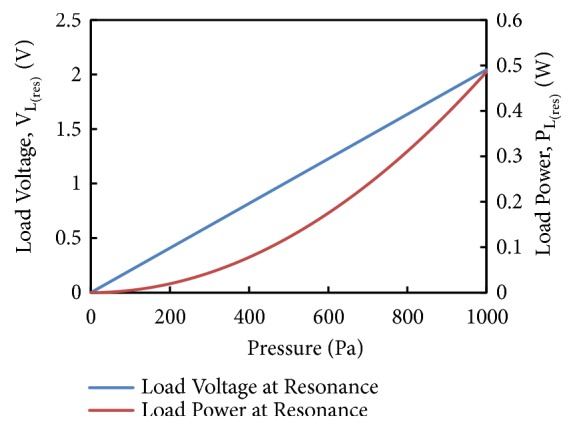
Load voltage and load power at resonance vs. amplitude of pulsating pressure.

Figure 12 shows the plot of load voltage and power at resonance as a function of pressure amplitude (0 to 1000 Pa) in the pipeline. The device's resonant frequency of 500 Hz and damping ratio *ξ*_*T*_ = 0.01 are considered in this computation (20). The simulations show that as the amplitude of the pulsating pressure is enhanced both voltage and power production increase; however, the voltage increase is linear in comparison to power. At pressure amplitude of 1000 Pa, a maximum load voltage of 2.04 V and maximum power of 0.49 W can be generated with device.

**Figure 13:**
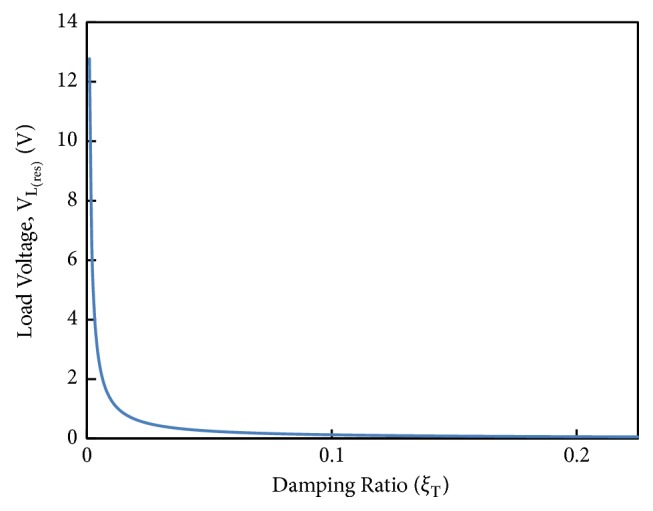
Load voltage at resonance vs. total damping ratio *ξ*_T_ of the harvester.

The simulation of the load voltage at resonance versus damping ratio is shown in Figure 13. The simulation reveals how the voltage generation can be affected with varying the damping (especially mechanical damping). The load voltage is computed at a resonant frequency of 500 Hz against the total damping ratio *ξ*_*T*_ ranging from 0.001 to 0.225. The trend shows that as the damping ratio increases the load voltage drops drastically. It means that as the damping in the harvester is reduced, the voltage generation will increase due to the increase in the amplitude of the relative velocity between magnet and coil. Practically this can be performed by reducing the mechanical damping in the device, such as packaging the harvester in vacuum or selecting such materials, for which material or support damping is minimal.

**Figure 14:**
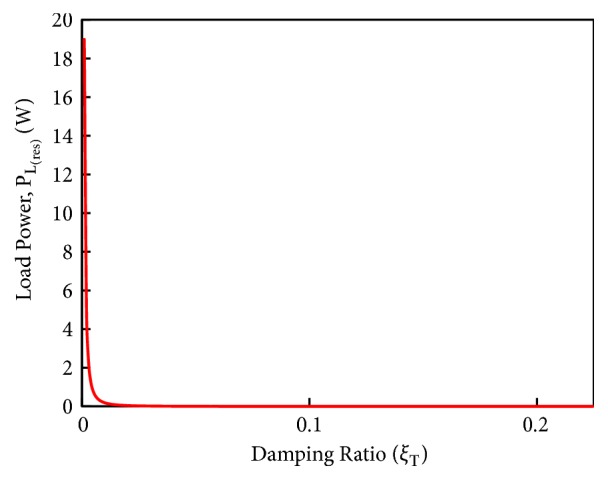
Load power at resonance vs. total damping ratio *ξ*_T_ of the harvester.

Figure 14 shows the load power achieved at resonance (500 Hz) as a function of total damping ratio *ξ*_*T*_. The simulation shows the decreasing trend of harvesters' power generation with increasing damping ratio. It shows the importance of total damping with regard to the harvester's performance; for example, for a harvester, in which *ξ*_*T*_ = 0.001, it is expected that with this harvester a power of 18.9 W can be easily produced. However, when the total damping in a harvester is relatively high, such as *ξ*_*T*_ = 0.01, the power production with that harvester will be 189.8 mW. Moreover, in contrast the total damping ratio of 0.1 actually leads to the power generation of only 1.898 mW.

## 4. Conclusions

An analytical model for a linear electromagnetic energy harvester operating with a pulsating fluid flow (in a pipeline) has been developed and simulations were performed as well. The harvester is modelled as a single degree of freedom system with linear spring and damper. The model for the relative velocity between the magnet and coil is utilized in Faraday's law of electromagnetism to obtain the analytical model for the voltage generation of the harvester. Moreover, for the multilayered wound coil of the harvester a simple analytical model is also devised in terms of coil's parameters. For the harvester, simulations are performed at a pressure of 625 Pa and total damping ratio of 0.01 at which the maximum open circuit voltage of 2.55 V is estimated at the resonant frequency of 500 Hz. Operated at resonance, the load voltage of 1.27 V is predicted at the optimum load resistance of 4.3 Ω. Moreover, the simulation results show a maximum power generation of 188151.2 *μ*W for the harvester. Furthermore, the simulations also reveal that as the damping in the harvester is reduced, the voltage and power generation will increase due to the enhancement in the amplitude of the relative velocity between magnet and coil. Practically this can be performed by reducing the mechanical damping in the device, such as packaging the harvester in vacuum or selecting such materials, for which material and support damping are minimal. In these harvesters it is also found out that the voltage and power production also depend on the amplitude of the pulsating pressure in the pipeline.

## Figures and Tables

**Table 1:** Pipeline condition monitoring.

Purpose	Country	Average length (km)	Monitored parameters	Monitoring system	Ref.
Water distribution	Saudi Arabia	4000	Pressure and flow rate	Pressure and flow monitoring system	[2]

Oil transportation	Alaska (TAPS)	1280	Pressure, flow rate, deviation, flow rate balance, and line volume balance	Seismic monitoring system and leak detection system	[3]

Water supply system	Goldfields Pipelines (Australia)	530	Pressure and flow velocity	Pressure and flow monitoring system	[4]

**Table 2:** Parameters and dimensions of the simulated energy harvester.

Parameters	Symbol	Dimensions
Height of the magnet	h_m_	22 mm

Radius of the magnet	r_m_	10 mm

Magnet type	NdFeB	-

Magnet's remnant flux density	B_r_	1.3 T

Height of the wound coil	h	3 mm

Resistance of the wound coil	R_C_	4.3 Ω

Mass of the coil	m	1.28 grams

Number of turns of the coil	n_t_	1400

Number of layers of the coil	N_L_	35

Number of turns per layer	n	40

Internal diameter of the coil	d_p_	5 mm

External diameter of the coil	d_e_	12 mm

Wire diameter	d_w_	100 *μ*m

Gap between magnet and coil	*z*	3 mm

Diameter of the membrane	D	20 mm

## Data Availability

The data [Table 2] used to support the findings of this study are included within the article.
